# Supplementary material for: The Functional Interaction of EGFR with AT1R or TP in Primary Vascular Smooth Muscle Cells Triggers a Synergistic Regulation of Gene Expression
Source: Cells. 2022 Jun 16;11(12):1936. doi: 10.3390/cells11121936 (PMC9222111; doi:10.3390/cells11121936)

SUPPLEMENTARY FIGURES

Figure S1 – Principal component analyses reveal a batch- and an animal-effect on gene expression

(a) All available data were first considered together (independent of the incubation time). Plotting the number of total raw counts per sample (each bar corresponds to one sample) showed a high variation in between the samples incubated for 48h, while this was not the case for the ones incubated for 24h. Proceeding with all samples together for further analysis steps would have led to overestimation and underestimation of the gene expression variations for the 24h and 48h data, respectively. Additionally, PCA performed on all samples did not allow to distinguish if the highest variance in between the sample groups (PC1) was induced by the incubation time or by the sequencing batches. For these reasons, the two datasets were analyzed separately. **(b)** Results of the analyses with DESeq2 and edgeR (FDR 0.05). The strategy to identify significantly differentially expressed genes (DEG) is shown for the comparison of EGF-treated vs. control group after 24h incubation. The genes found to be regulated by both tools were selected and their *log2* fold changes calculated by DESeq2 and edgeR appeared highly correlated. The genes were finally filtered for “abundance” and “consistency”. The remaining genes were defined as DEG. **(c,d)** PCA was performed during both analysis rounds, for data after **(c)** 24h and **(d)** 48h incubation. Principal components 1 and 2 (PC1 and PC2) are displayed here. For each time-point, PC1 appears to correspond to the animal from which aVSMC were isolated. The variance induced by the incubation type underlines PC2. Therefore, the multi-variable design ~ animal + treatment was used for both DESeq2 and edgeR.

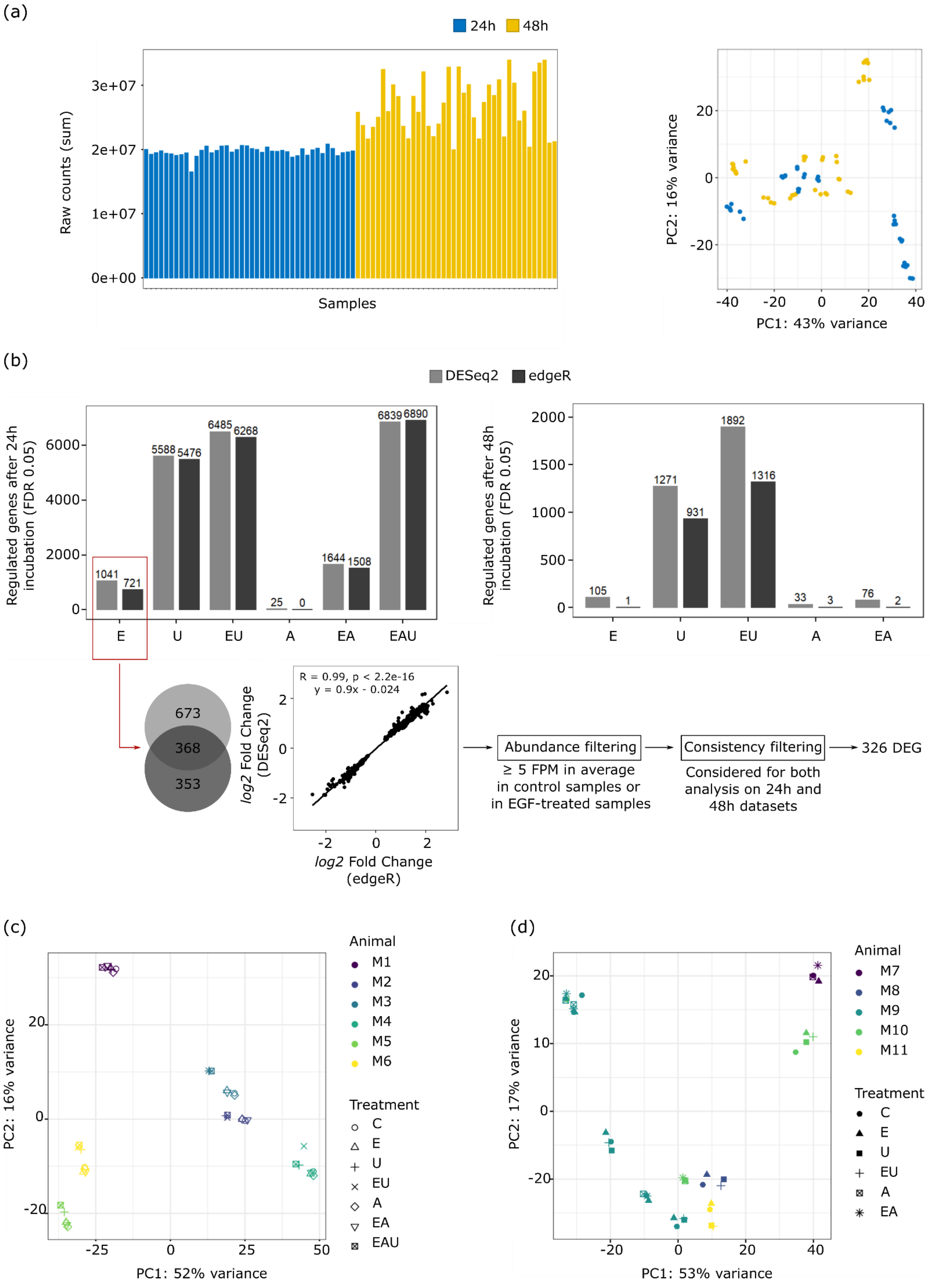

(a)
24h
48h
Raw counts (sum)
3e+07
2e+07
1e+07
0e+00
Samples
PC2: 16% variance
PC1: 43% variance
(b)
DESeq2
edgeR
Regulated genes after 24h incubation (FDR 0.05)
1041
721
5588
5476
6485
6268
25
0
1644
1508
6839
6890
E
U
EU
A
EA
EAU
Regulated genes after 48h incubation (FDR 0.05)
105
1
1271
931
1892
1316
33
3
76
2
673
368
353
R = 0.99, p < 2.2e-16
y = 0.9x - 0.024
log2 Fold Change (DESeq2)
log2 Fold Change (edgeR)
Abundance filtering
≥ 5 FPM in average in control samples or in EGF-treated samples
Consistency filtering
Considered for both analysis on 24h and 48h datasets
326 DEG
(c)
PC2: 16% variance
PC1: 52% variance
Animal
M1
M2
M3
M4
M5
M6
Treatment
C
E
U
EU
A
EA
EAU
(d)
PC2: 17% variance
PC1: 53% variance
Animal
M7
M8
M9
M10
M11
Treatment
C
E
U
EU
A
EA

Figure S2 – aVSMC express genes coding for AT1R, EGFR and TP but respond to exclusive EGF- and U46619-stimulation only

(a) FPM for the genes coding for each receptor and GAPDH in control samples (24h and 48h incubation). The dotted line corresponds to the 5 FPM threshold employed to filter out lowly expressed genes. **(b)** The mRNA abundance was measured by ddPCR for each receptor (N = 4). **(c)** *Tbxa2r* mRNA expression after 24h, measured by ddPCR. The control group was used as reference. (N =4) **(d)** EGFR relative protein expression after 24h and 48h incubation (N =6) **(e)** The amount of phosphorylated EGFR (N = 4) and the ratio of phosphorylated ERK1/2 / total ERK1/2 (N = 5) were measured by estern Blot. “P” stands for PMA-treated sampled, used here as positive control for ERK1/2 phosphorylation. (N = 5) (* $p < 0.05$)

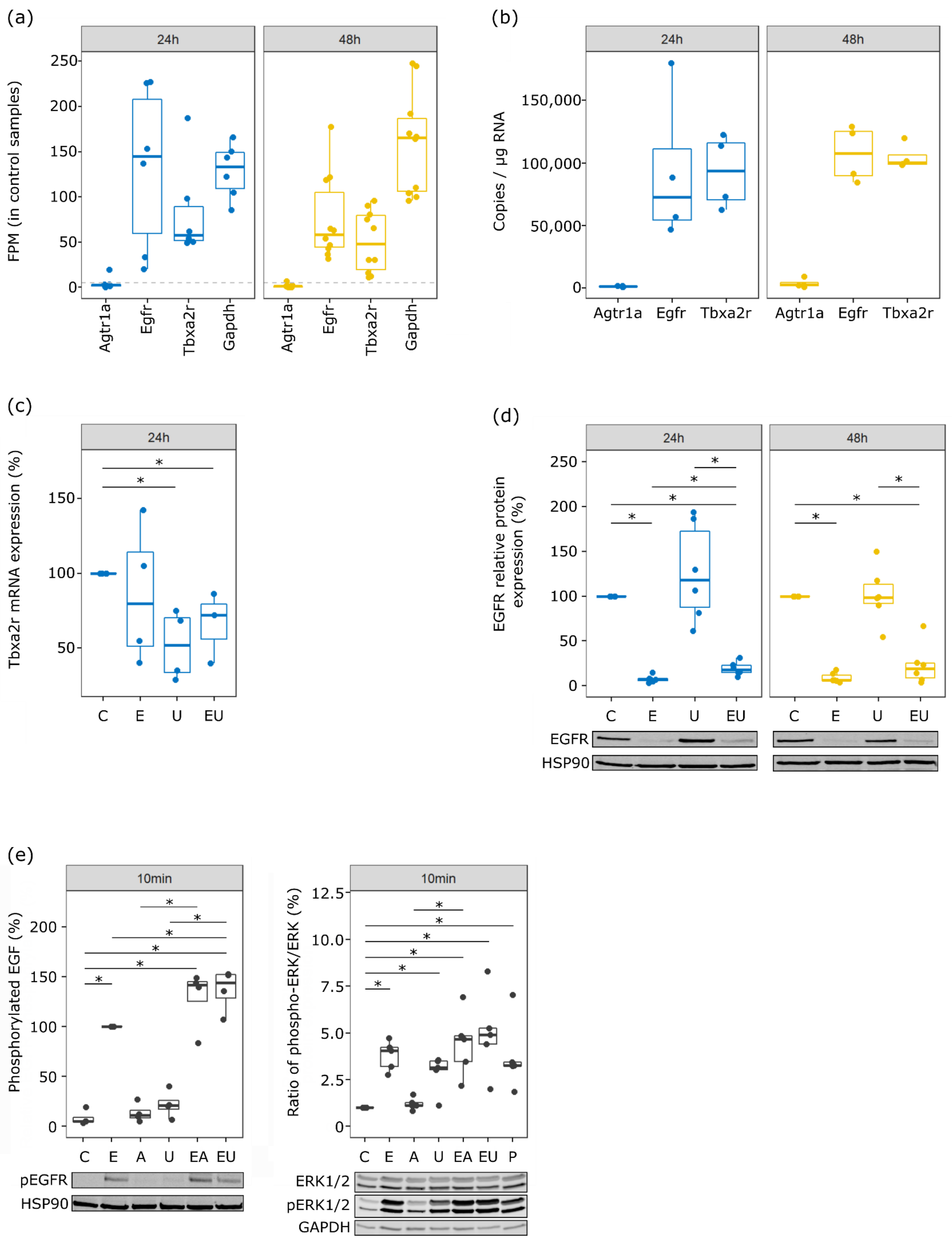

(a)
24h
48h
FPM (in control samples)
250
200
150
100
50
0
Agtr1a
Egfr
Tbxa2r
Gapdh
(b)
24h
48h
Copies / µg RNA
150,000
100,000
50,000
0
Agtr1a
Egfr
Tbxa2r
(c)
24h
Tbxa2r mRNA expression (%)
150
100
50
C
E
U
EU
(d)
24h
48h
EGFR relative protein expression (%)
250
200
150
100
50
0
C
E
U
EU
EGFR
HSP90
(e)
10min
Phosphorylated EGF (%)
200
150
100
50
0
C
E
A
U
EA
EU
pEGFR
HSP90
10min
Ratio of phospho-ERK/ERK (%)
12.5
10.0
7.5
5.0
2.5
0
C
E
A
U
EA
EU
P
ERK1/2
pERK1/2
GAPDH

Figure S3 – Differentially expressed genes clustering

As complement to Figure 1. Heatmaps showing the normalized expression (*log* scale, calculated with rlog function from DESeq2) of genes identified as significantly regulated for at least one comparison (treated vs. control group), in the analyses for 24h **(a)** or 48h **(b)** incubation. Each row represents a gene and each column a sample. Expression levels were additionally row-wised centered (substraction of the mean to each values) and scaled (division by the standard deviation). Rows were clustered based on Euclidean distance (complete method, calculated by pheatmap - https://cran.r-project.org/package=pheatmap).

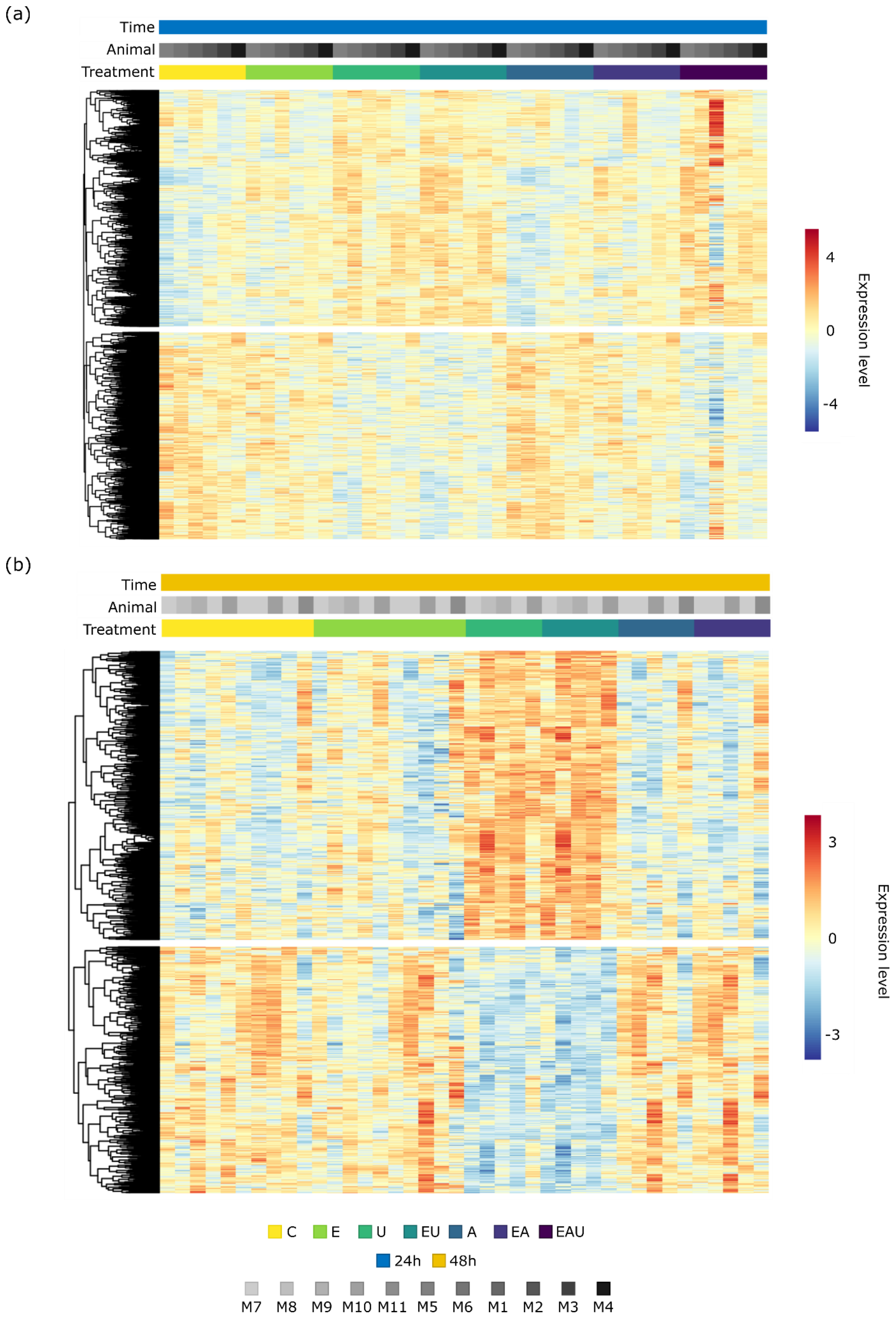

(a)
Time
Animal
Treatment
4
0
-4
Expression level
(b)
Time
Animal
Treatment
3
0
-3
Expression level
C
E
U
EU
A
EA
EAU
24h
48h
M7
M8
M9
M10
M11
M5
M6
M1
M2
M3
M4

Figure S4 – log2 fold change distributions of DEG after 24h incubation

Histograms and corresponding density curves of *log2* fold change (calculated by edgeR) of the genes included in intersections UpSet plot based on results after 24h incubation (Figure 2a). Computed only for intersections with more than 50 genes (i1 to i10). Kolmogorov-Smirnov test was used to assess if the shifts in distribution were statistically significant. Significant p-values ($p < 0.05$) and the corresponding comparisons are indicated.

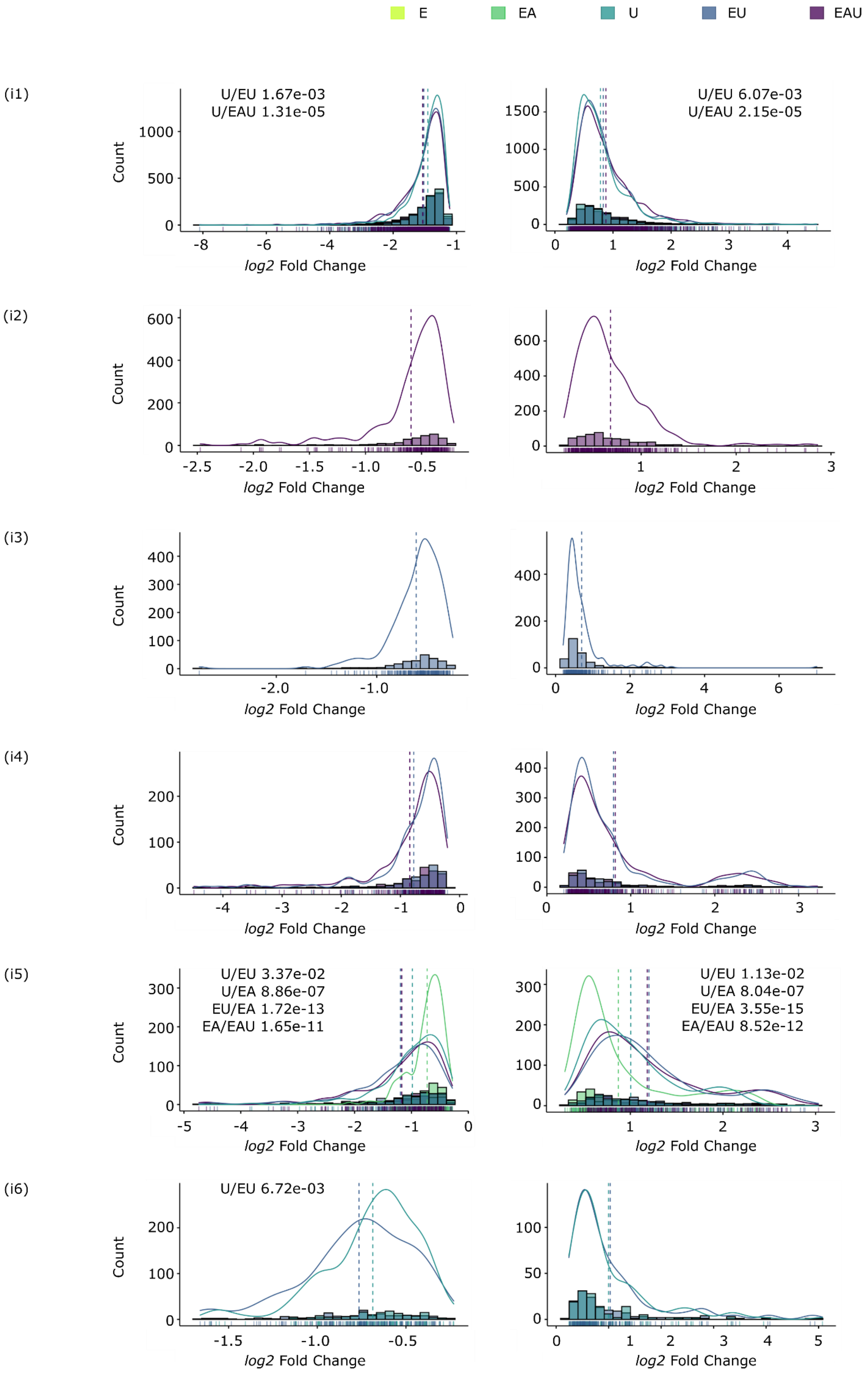
E
EA
U
EU
EAU
(i1)
U/EU 1.67e-03
U/EAU 1.31e-05
U/EU 6.07e-03
U/EAU 2.15e-05
(i2)
(i3)
(i4)
(i5)
U/EU 3.37e-02
U/EA 8.86e-07
EU/EA 1.72e-13
EA/EAU 1.65e-11
U/EU 1.13e-02
U/EA 8.04e-07
EU/EA 3.55e-15
EA/EAU 8.52e-12
(i6)
U/EU 6.72e-03
Count
log2 Fold Change

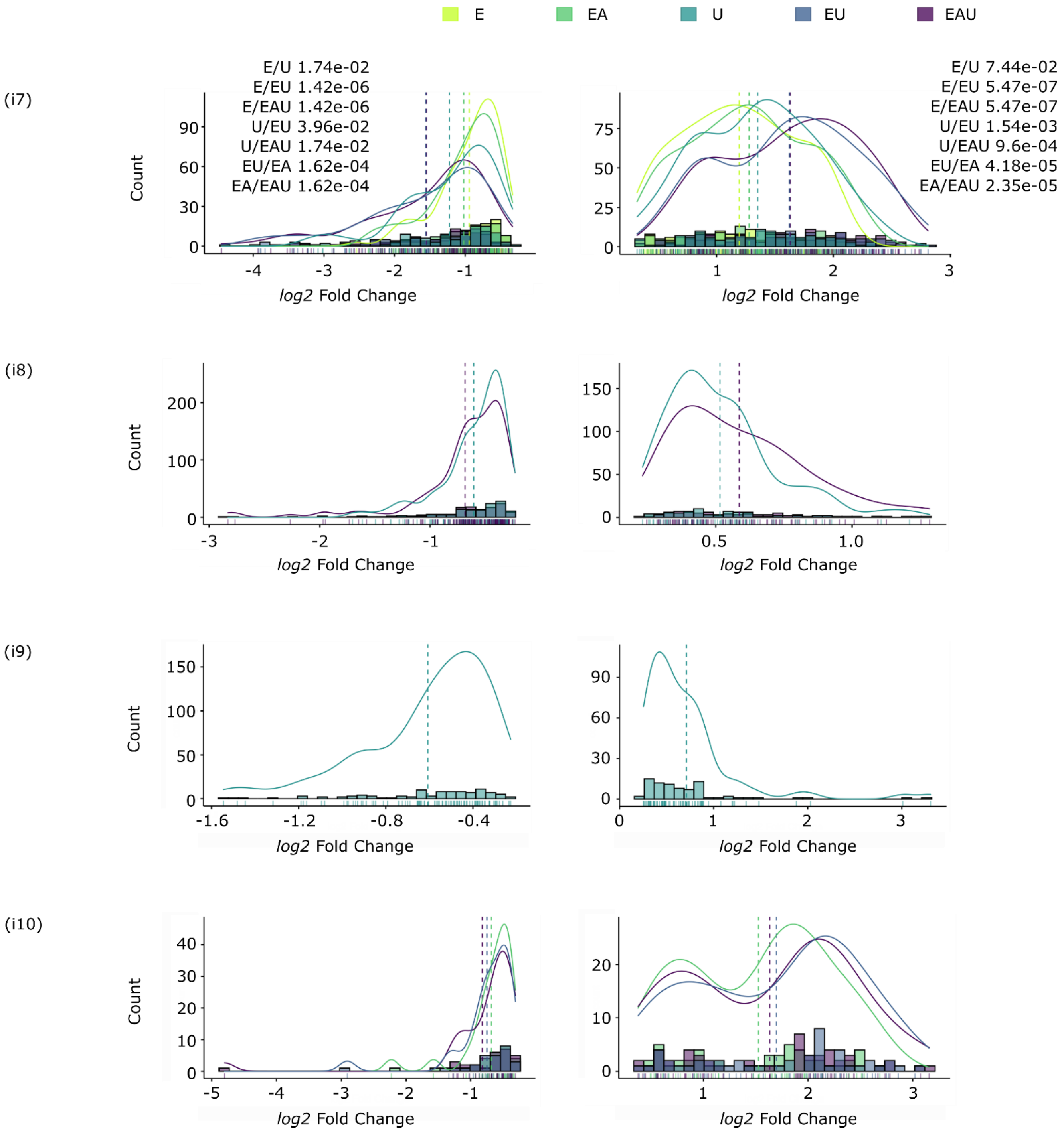
E
EA
U
EU
EAU
(i7)
E/U 1.74e-02
E/EU 1.42e-06
E/EAU 1.42e-06
U/EU 3.96e-02
U/EAU 1.74e-02
EU/EA 1.62e-04
EA/EAU 1.62e-04
E/U 7.44e-02
E/EU 5.47e-07
E/EAU 5.47e-07
U/EU 1.54e-03
U/EAU 9.6e-04
EU/EA 4.18e-05
EA/EAU 2.35e-05
(i8)
(i9)
(i10)
Count
log2 Fold Change

Figure S5 – Overview of the upstream regulator analysis results

Original figure produced by IPA Software. Hierarchical clustering of the predicted regulators (filtered for “Gene, RNA, proteins”) that had a |Z-score| ≥ 2 and a –log(adjusted p-value) ≥ 3 in at least one of the three compared analyses (control vs. EGF or vs. U46619 or vs. EGF and U46619). They were filtered in 3 categories: EGF-specific (∗), U46619-specific (#) or EGF and U46619-specific (+) (Table 2).

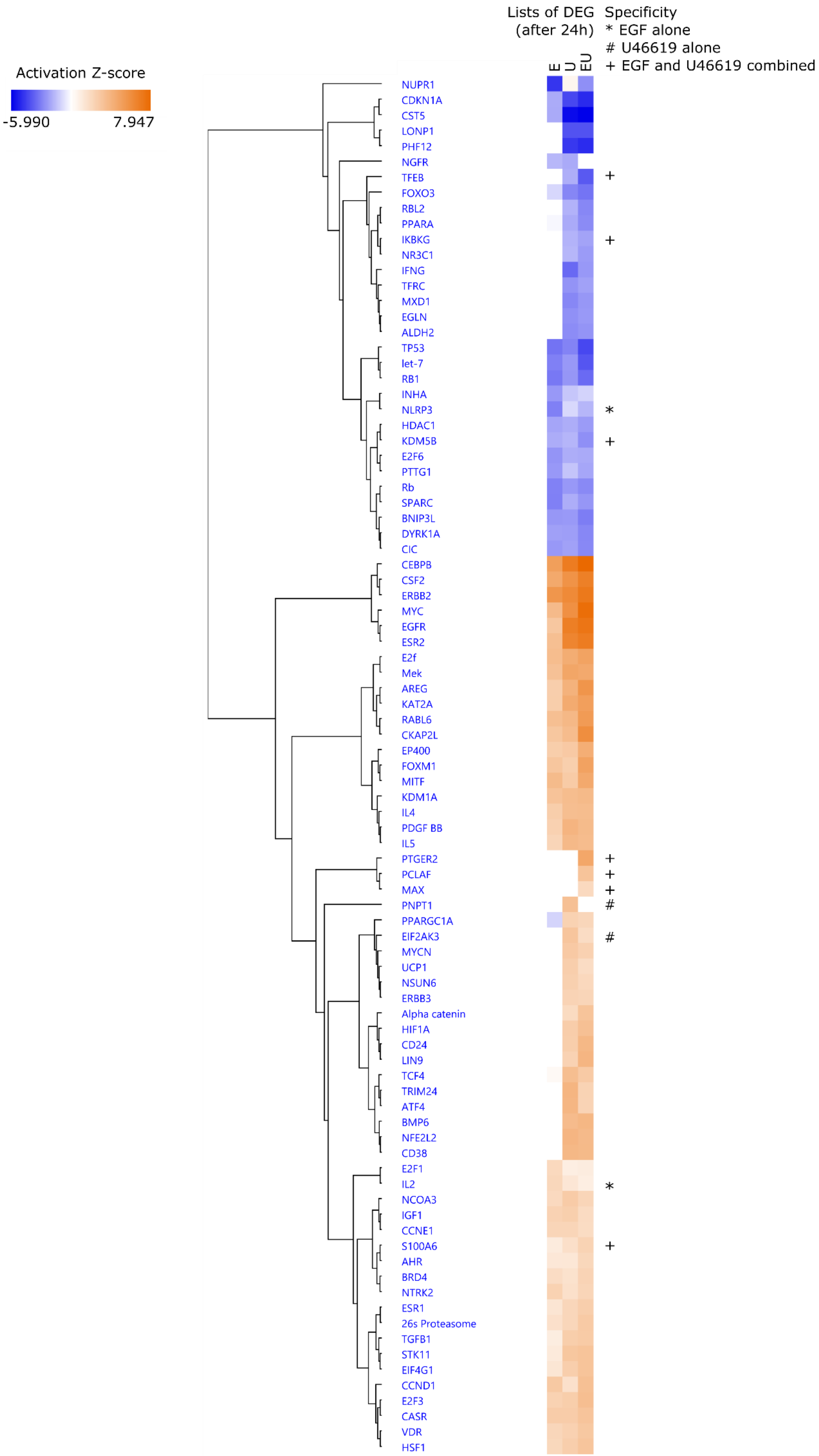

Activation Z-score
-5.990
7.947
Lists of DEG (after 24h)
E
U
EU
Specificity
* EGF alone
# U46619 alone
+ EGF and U46619 combined
NUPR1
CDKN1A
CST5
LONP1
PHF12
NGFR
TFEB +
FOXO3
RBL2
PPARA
IKBKG +
NR3C1
IFNG
TFRC
MXD1
EGLN
ALDH2
TP53
let-7
RB1
INHA
NLRP3 *
HDAC1
KDM5B +
E2F6
PTTG1
Rb
SPARC
BNIP3L
DYRK1A
CIC
CEBPB
CSF2
ERBB2
MYC
EGFR
ESR2
E2f
Mek
AREG
KAT2A
RABL6
CKAP2L
EP400
FOXM1
MITF
KDM1A
IL4
PDGF BB
IL5
PTGER2 +
PCLAF +
MAX +
PNPT1 #
PPARGC1A
EIF2AK3 #
MYCN
UCP1
NSUN6
ERBB3
Alpha catenin
HIF1A
CD24
LIN9
TCF4
TRIM24
ATF4
BMP6
NFE2L2
CD38
E2F1
IL2 *
NCOA3
IGF1
CCNE1
S100A6 +
AHR
BRD4
NTRK2
ESR1
26s Proteasome
TGFB1
STK11
EIF4G1
CCND1
E2F3
CASR
VDR
HSF1

Figure S6 - U46619 itself or combined with EGF regulate gene expression in the same direction after 24h and 48h incubation

Scatter plots of the *log2* fold changes (computed by edgeR) of the DEG comprised in the overlaps of the results of 24h and 48h analyses for U46619 and "EGF and U46619" incubations (Figure 3b).

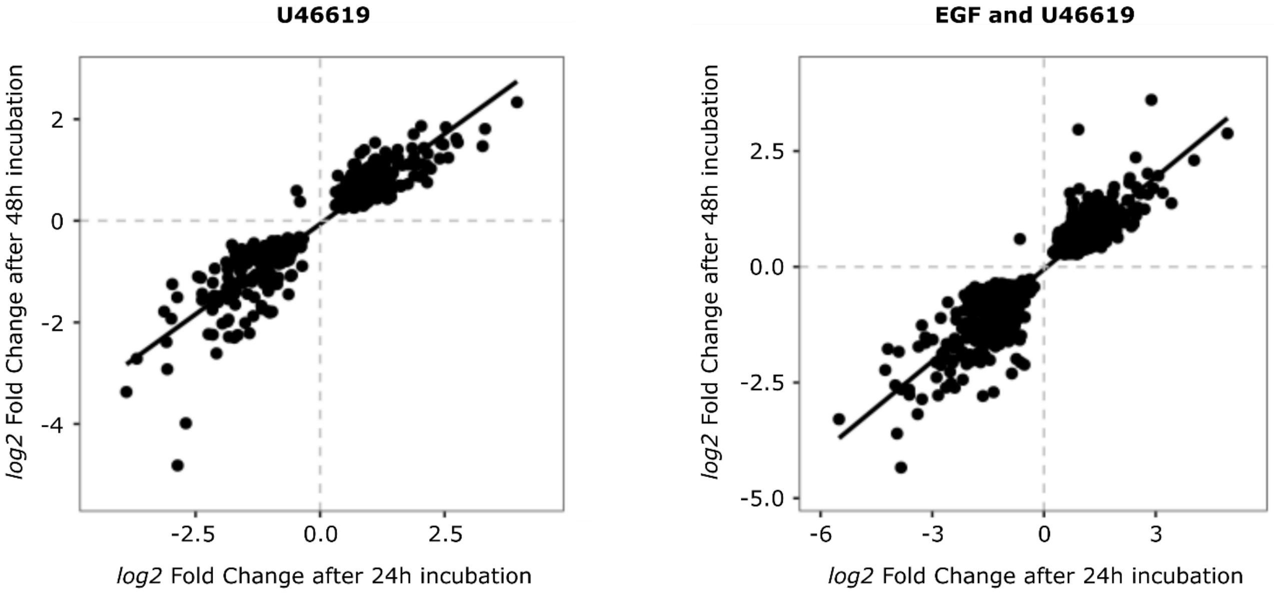

Figure S7 – FPM comparison to highlight synergistic regulation by EGF with U46619 or AngII

Filtering for genes with significantly stronger regulation by combined incubations, based on FPM comparison (listed in Table 3). The dotted line corresponds to the 5 FPM threshold employed to filter out lowly expressed genes. (* $p < 0.05$, *t*-test)

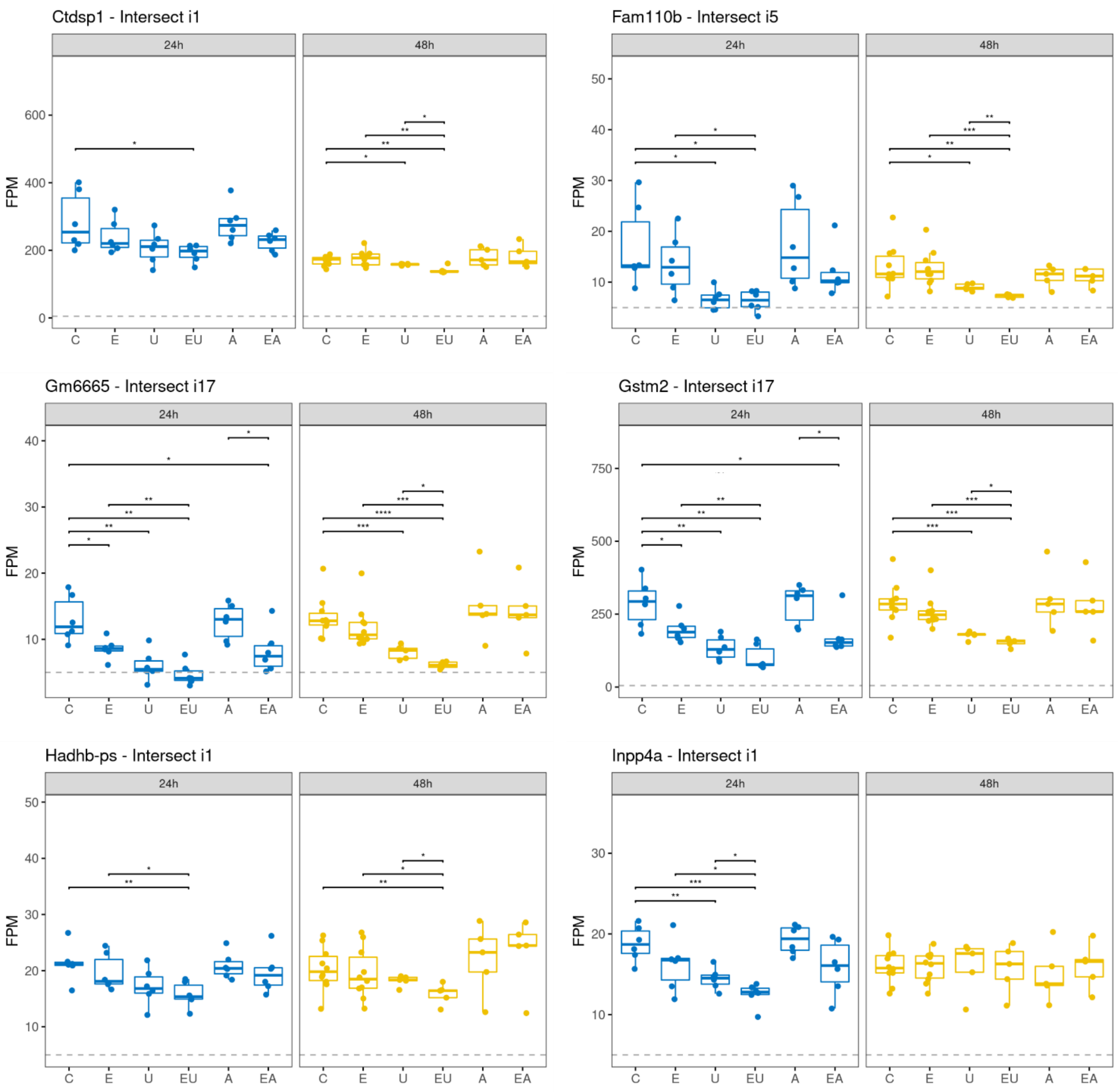

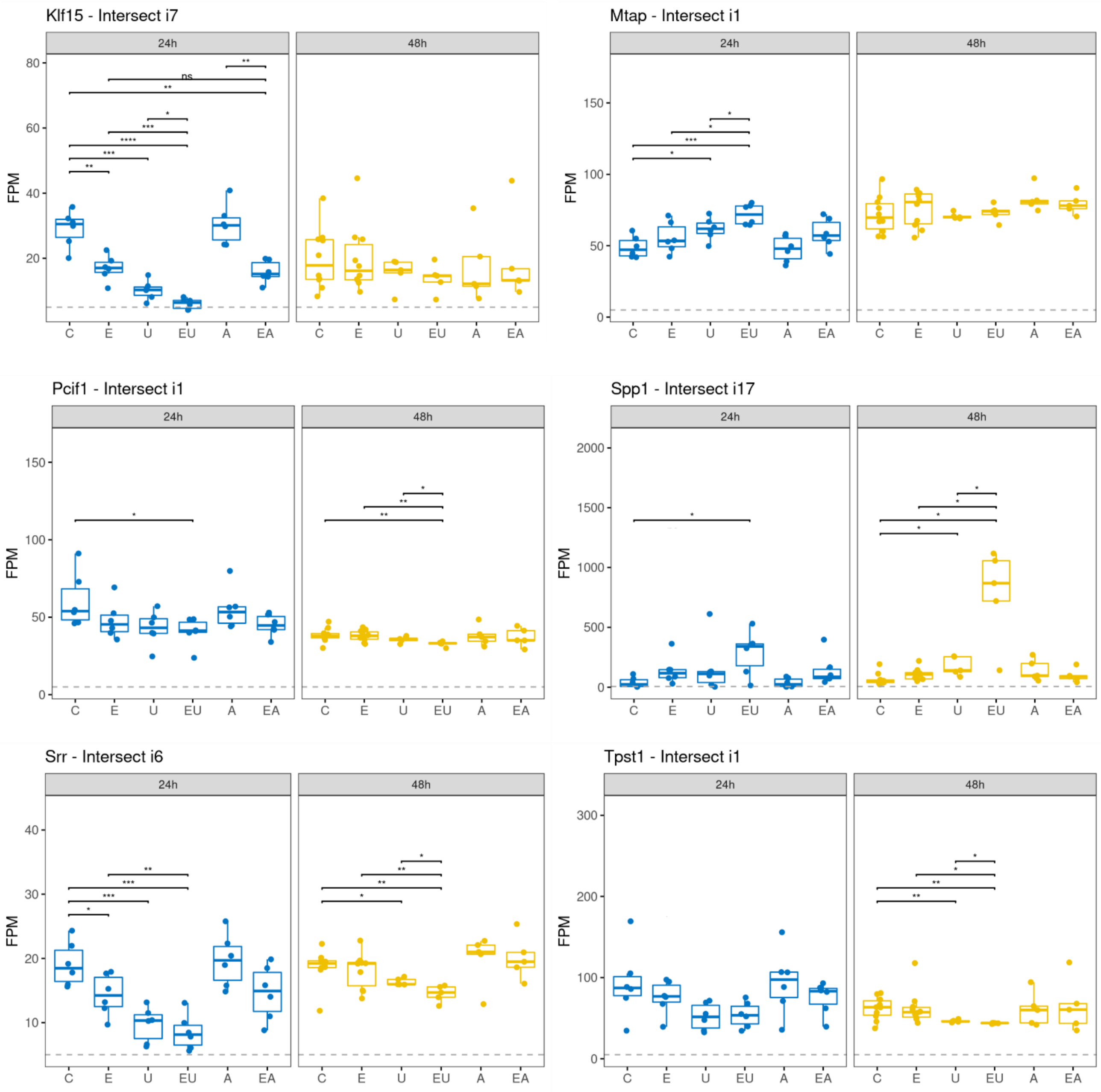

Klf15 - Intersect i7
Mtap - Intersect i1
Pcif1 - Intersect i1
Spp1 - Intersect i17
Srr - Intersect i6
Tpst1 - Intersect i1
24h
48h
FPM
C
E
U
EU
A
EA

Figure S8 – Western Blot membranes

Original pictures of the membranes considered for the quantification of EGFR, pEGFR, (p)ERK (Results shown in Supplementary Fig. 2d and 2e). Membranes were detected with an Odyssey imaging system (LI-COR Biosciences) that allows the simultaneous detection of secondary antibodies in two fluorescent channels (red and green). Membranes were were cut prior to the incubation with primary antibodies (upper part with (p)EGFR and HSP90, lower part with (p)ERK and GAPDH).

Western Blot
10min incubation (Fig. S2e)

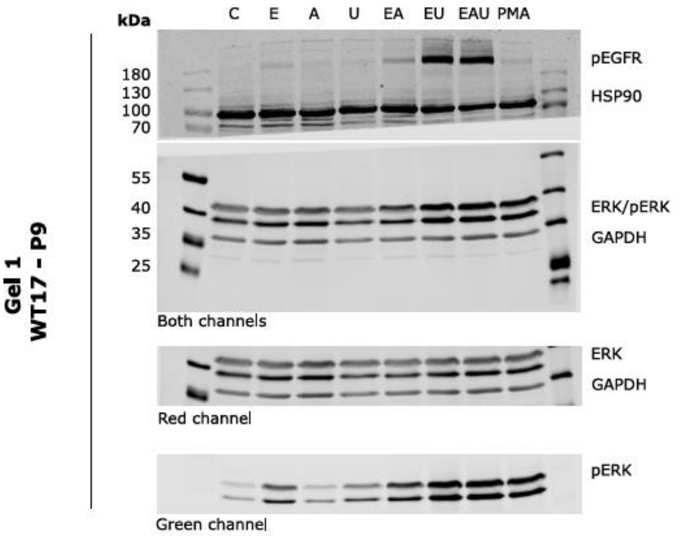

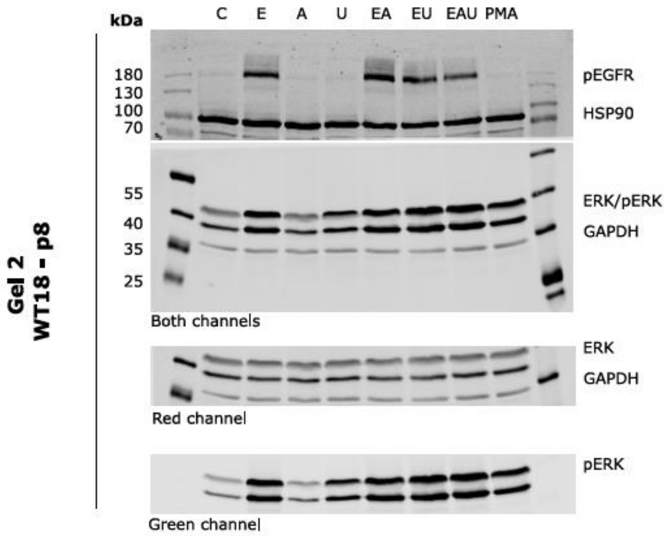

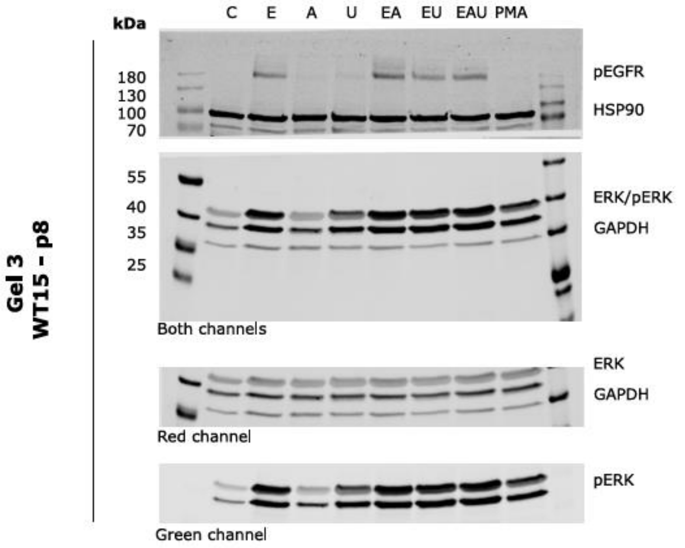

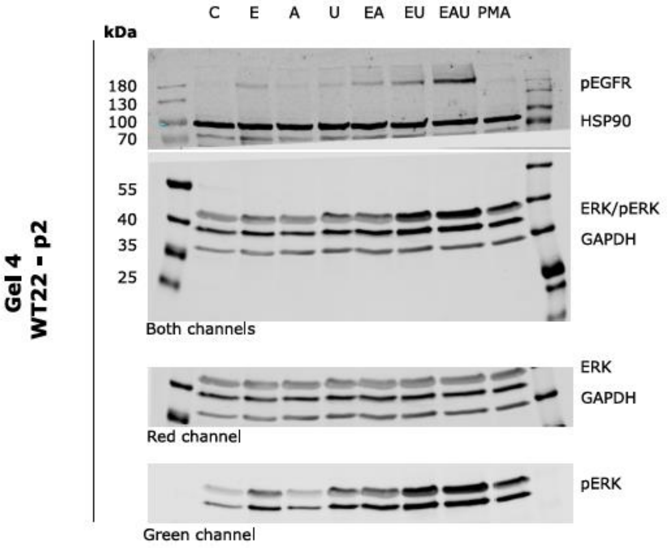

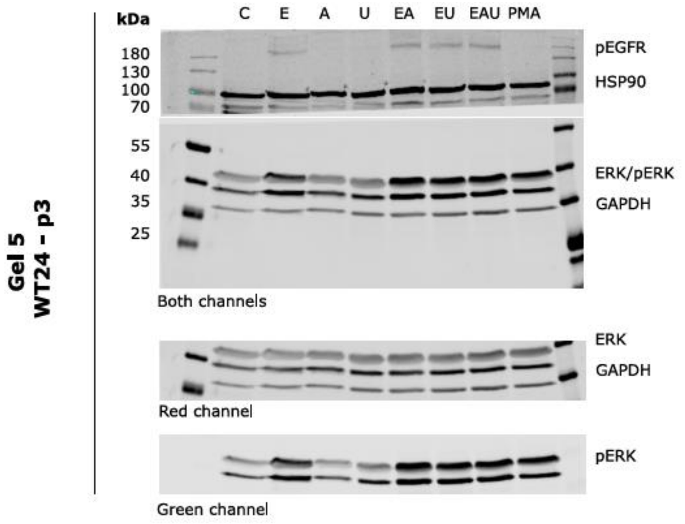

Western Blot
24h/48h incubation (Fig. S2d)

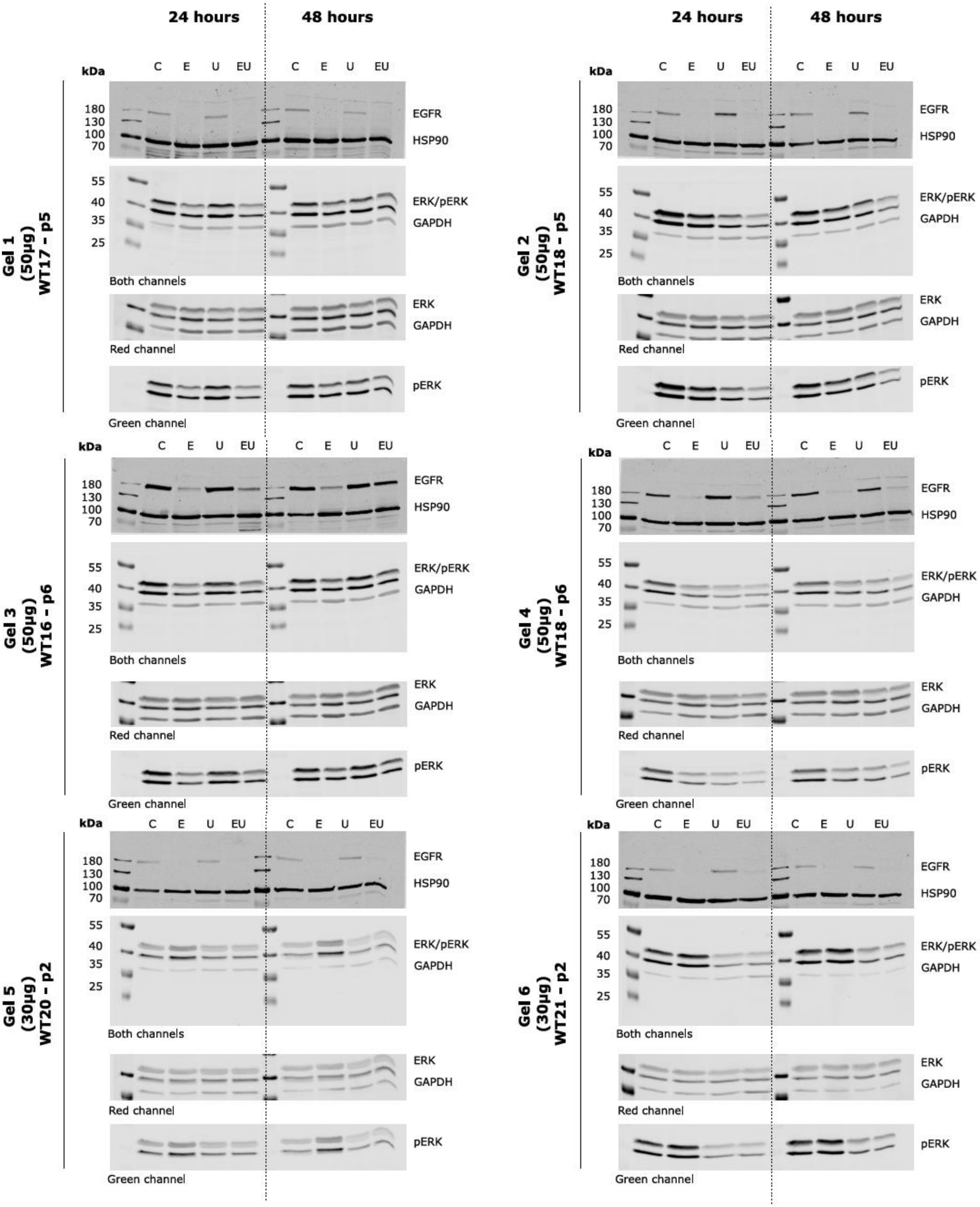

Supplement: Supplementary file 1 [file cells-11-01936-s001.zip › Supplementary Figures.pdf]
