# Supplementary material for: The Functional Interaction of EGFR with AT1R or TP in Primary Vascular Smooth Muscle Cells Triggers a Synergistic Regulation of Gene Expression
Source: Cells. 2022 Jun 16;11(12):1936. doi: 10.3390/cells11121936 (PMC9222111; doi:10.3390/cells11121936)
Supplement: Supplementary file 1 [file cells-11-01936-s001.zip › Supplementary Methods.pdf]

SUPPLEMENTARY METHODS

qPCR and ddPCR primer sequences

Gene	Sense (5' to 3')	Antisense (5' to 3')	Annealing temp. (°C)
Gapdh	TTGAGGTCAATGAAGGGGTC	TCGTCCCGTAGACAAAATGG	62
Eef2	TTCTGATGATGGGCCGCTAC	AAGGTAGTGATGGTCCCCGT	60
Spp1	ATTTGCTTTTGCCTGTTTGG	TGGCTATAGGATCTGGGTGC	57
Klf15	TCCTCCAACTTGAACCTGCC	GTGGGAAGCGATGCACTTTG	62
Egfr	GACCTTCACATCCTGCCAGT	GCATGGAGGTCAGTCCAGTT	63
At1ra	AAGGGCCATTTTGCTTTTCT	AATGGCTGGCATTTTGTCTG	60
Tbxa2r	GTGTGTTGGATGCCCTTGCT	CCACACGCAGGTAGATGAGC	64.6

Antibodies

Target antigen	Source	Catalog #	Working conc.
EGFR	Cell Signaling Technology	4267	1:1 000
Phosphorylated EGFR (Y1068)	Cell Signaling Technology	3777	1:1 000
ERK1/2	Cell Signaling Technology	4696	1:1 000
Phosphorylated ERK1/2	Cell Signaling Technology	9101	1:1 000
GAPDH	Cell Signaling Technology	97166	1:1 000
HSP90	Cell Signaling Technology	4874	1:1 000
Anti-Mouse IRDye 680RD	LICOR Biosciences	926-68070	1:20 000
Anti-Rabbit IRDye 800CW	LICOR Biosciences	926-32211	1:20 000
BrdU	Becton Dickinson	347580	1:2 000
Anti-mouse IgG, HRP-linked	Cell Signaling Technology	7076	1:1 000

Buffer compositions

Buffer name	Composition
Blocking Solution	0.1% Triton X-100 in PBS, 10% FCS

Caspase-reaction buffer	20 mM PIPES, 4 mM EDTA, 0.2% CHAPS, 10 mM DTT, pH 7.4
Cell lysis buffer (for caspase3 activity)	20 mM MOPS, 0.1% Triton X-100, pH 7.4 NaOH
HEPES-Ringer buffer	122.5 mM NaCl, 5.4 mM KCl, 0.8 mM MgCl2·6H2O, 1.2 mM CaCl2·2H2O, 1 mM NaH2PO4·H2O, 5.5 D-glucose, 10 mM HEPES, pH 7.4
HRP-substrate	11.8 mg/mL Sodium phosphate dibasic dehydrate, 7.3 mg/mL Citric Acid, 0.5 mg/mL o-Phenylenediamine, 0.015% H2O2
Laemmli Buffer	0.5 M Tris pH 6.8, 10% SDS, 10% Gylcerol
Permabilization Buffer	0.1% Triton X-100 in PBS, 37 mg/L Na-Orthovanadate
RIPA buffer	150 mM NaCl, 10 mM Tris-base, 1% Nonidet P-40, 0.1% SDS, 1% Na-Deoxycholate, 0.1% Triton X-100, 1:500 Protease Inhibitor Cocktail, 1 mM EDTA, 184 mg/L Na-Orthovanadate, pH 7.4
TBS	17 mM Tris-HCl, 3 mM Tris-base, 140 mM NaCl, pH 7.4 HCl
TBS-Tween	TBS with 0.1% Tween-20

Chemical and kit providers

Chemical	Provider
AFC	Sigma, Germany
Angiotensin II	Sigma, Germany
BCA assay reagents	ThermoScientific, Germany
BlueZol	Serva, Germany
BrdU	Sigma, Germany
C_{12}FDG	ThermoFisher Scientific, Germany
DEVD-AFC	AAT Bioquest, Biomol, Germany
DMEM media, low glucose media powder	Sigma, Germany (#D5523)
EGF	Merck, Germany
FCS	Pan Biotech, Germany
Hoechst 33342	ThermoFisher Scientific, Germany
Hydrocortisone	Sigma, Germany
Insulin	Eli Lilly and Company, Germany
PlatinumTM SYBRTM Green	Invitrogen, Life Technologies, Germany
PMA	Sigma, Germany
Protease inhibitor	Sigma, Germany (#P8340)
Sodium selenite	Sigma, Germany

SuperScript II reverse transcriptase	Invitrogen, Life Technologies, Germany
Transferrin	Sigma, Germany
Trypan Blue	Life Technologies, Germany
Turbo DNAse-free kit	Invitrogen, Life Technologies, Germany
U46619	Bio-Techne, Germany
**Kit**	**Provider**
QX200 ddPCR EvaGreen Supermix	BioRad, Germany
Mouse Osteopontin ELISA kit	Sigma, Germany (RAB0437)

Abbreviations within Methods

AFC	7-amino-4-trifluoromethylcoumarin
BSA	Bovine serum albumin
C_{12}FDG	5-Dodecanoylaminofluorescein Di-β-D-Galactopyranoside
$CaCl_2 \cdot 2H_2O$	Calcium chloride dihydrate
CHAPS	3-[(3-cholamidopropyl)dimethylammonio]-1-propanesulfonate
CST	Cell Signaling Technologies
DMEM	Dulbecco’s Modified Eagle Medium
ddPCR	Digital droplet PCR
DEG	Differentially expressed genes
DTT	Dithiothreitol
EDTA	Ethylenediaminetetraacetic acid
ELISA	Enzyme-linked immunosorbent assay
FC	Fold change
FCS	Fetal calf serum
HCl	Hydrogen chloride
HEPES	4-(2-hydroxyethyl)-1-piperazineethanesulfonic acid
HRP	Horseradish peroxidase
IgG	Immunoglobulin G
KCl	Potassium chloride

$MgCl_2 \cdot 6H_2O$	Magnesium chloride hexahydrate
MOPS	3-(N-morpholino)propanesulfonic acid
NaCl	Sodium chloride
$NaH_2PO_4 \cdot H_2O$	Sodium phosphate monobasic monohydrate
$NaHCO_3$	Sodium bicarbonate
PBS	Phosphate-buffered saline
PCA	Principal Component Analysis
PDGF	Platelet-derived growth factor
PIPES	Piperazine-N,N′-bis(2-ethanesulfonic acid)
PMA	Phorbol 12-myristate 13-acetate
qPCR	Quantitative PCR
RIN	RNA integrity number
RT°	Room temperature
SDS-PAGE	Sodium dodecyl sulfate-polyacrylamide gel electrophoresis
TBS	Tris-buffered saline
TMM	Trimmed mean of M value
